# Identifying predictors of adverse outcomes after termination of seclusion in psychiatric intensive care units

**DOI:** 10.1192/bjo.2024.710

**Published:** 2024-05-22

**Authors:** Jonathan P. Rogers, Gabriella Lewis, Maria Lobo, Clementine Wyke, Alexander Meaburn, Fiona Harding, Rebecca Garvey, Jenny Irvine, Ahmed Saeed Yahya, Daisy Kornblum, Alexis E. Cullen, David Mirfin, Glyn Lewis

**Affiliations:** Division of Psychiatry, University College London, UK; South London and Maudsley NHS Foundation Trust, London, UK; Priory Hospital North London, London, UK; Department of Clinical Neuroscience, Karolinska Institutet, Stockholm, Sweden; Department of Psychosis Studies, Institute of Psychiatry, Psychology and Neuroscience, King's College London, UK

**Keywords:** Seclusion, psychiatric intensive care unit, violence, aggression

## Abstract

**Background:**

Seclusion is a restrictive practice that many healthcare services are trying to reduce. Previous studies have sought to identify predictors of seclusion initiation, but few have investigated factors associated with adverse outcomes after seclusion termination.

**Aims:**

To assess the factors that predict an adverse outcome within 24 h of seclusion termination.

**Method:**

In a cohort study of individuals secluded in psychiatric intensive care units, we investigated factors associated with any of the following outcomes: actual violence, attempted violence, or reinitiation of seclusion within 24 h of seclusion termination. Among the seclusion episodes that were initiated between 29 March 2018 and 4 March 2019, we investigated the exposures of medication cooperation, seclusion duration, termination out of working hours, involvement of medical staff in the final seclusion review, lack of insight, and agitation or irritability. In a mixed-effects logistic regression model, associations between each exposure and the outcome were calculated. Odds ratios were calculated unadjusted and adjusted for demographic and clinical variables.

**Results:**

We identified 254 seclusion episodes from 122 individuals (40 female, 82 male), of which 106 (41.7%) had an adverse outcome within 24 h of seclusion termination. Agitation or irritability was associated with an adverse outcome, odds ratio 1.92 (95% CI 1.03 to 3.56, *P* = 0.04), but there was no statistically significant association with any of the other exposures, although confidence intervals were broad.

**Conclusions:**

Agitation or irritability in the hours preceding termination of seclusion may predict an adverse outcome. The study was not powered to detect other potentially clinically significant factors.

The definition of seclusion varies between organisations and in different countries around the world; however, it is generally agreed that seclusion constitutes the confinement of a patient in a space separate from other patients, where the individual is physically prevented from leaving either by staff or by physical barriers such as a locked door.^1^ In recent decades, mental health services around the world have moved towards a more patient-centred approach, with human rights and collaborative care at the forefront, and restrictive practice minimised where possible.^2^ Particular concerns have been raised regarding the risks to physical health associated with coercive practice,^3^ and the potential influence of non-clinical factors such as country,^4^ institutional culture,^5^ available facilities,^6^ nursing characteristics^7^ and ethnicity^8^ on seclusion rates.

Many interventions have been developed to reduce the use of seclusion, including environmental interventions, staff training, treatment planning, risk assessment, therapeutic activities, alternative responses to individual behaviour, sensory modulation, and individual and family involvement, with varying success.^9^ Few studies have focused on the duration of seclusion episodes, and the median reported duration varies greatly from less than 6 to more than 200 h.^10,11^ Factors such as male gender, psychotic diagnosis, hallucinations, and seclusion initiation due to risk of harm to others are associated with longer duration of seclusion.^11,12^ Female gender and diagnosis of drug-induced psychosis are associated with shorter duration of seclusion episodes,^13^ as are interventions such as administration of medication during the seclusion period and collaborative goal setting with individuals.^4^

Official guidance on the process of a seclusion review is notably absent, although one paper recommends a five-stage process including information gathering, mental state examination, assessment of physical health, risk assessment and debrief.^14^ Unfortunately, adverse outcomes sometimes occur after terminating seclusion: sometimes aggression subsequently escalates and seclusion is reinitiated. There is currently no systematic evidence on what factors may increase the risk of adverse outcomes after seclusion termination, although patient debriefing and step-down management planning^14^ have been suggested as methods to reduce this risk.

The aim of this study was to examine the predictors of adverse outcomes after seclusion termination (as indicated by seclusion reinitiation, actual violence or attempted violence) in adults in psychiatric intensive care units (PICUs). Given the paucity of the existing evidence, our hypotheses are mostly based on the broader literature around seclusion and restrictive practices. In terms of staffing factors, there is evidence that staffing levels and confidence within a team affect decision-making in terms of initiating and terminating seclusion.^15–17^ Staffing levels – particularly of senior staff – tend to be reduced out of hours, and seclusion episodes that are initiated during the weekend tend to be longer,^18^ so it is plausible that this would impact the success of seclusion termination. Given that medical staff have been viewed as having a distinctive role in the process of seclusion,^19^ and the presence of a senior doctor is associated with the termination of seclusion,^18^ we speculated that involving doctors in decision-making may also affect the success of seclusion termination.

In terms of patient factors, qualitative research has suggested that ward staff place an emphasis on people cooperating with their care when deciding whether to terminate seclusion.^16^ One of the more measurable manifestations of this is medication cooperation. Regarding psychopathology, aggression and agitation have been identified as being predictive of seclusion initiation.^20,21^ A lack of insight has also previously been found to predict seclusion initiation, and one guideline has highlighted the importance of assessing the individual's insight into the reasons for seclusion when deciding whether to terminate seclusion.^14^

Therefore, we wished to test the hypotheses that the following factors were associated with a higher risk of an adverse outcome within 24 h of terminating seclusion:

- incomplete medication cooperation during seclusion
- shorter duration of seclusion
- seclusion termination outside of working hours
- final seclusion review not involving medical staff
- lack of insight into the reasons for seclusion
- agitation or irritability.

## Method

### Study design

This retrospective cohort study is described according to the Strengthening the Reporting of Observational Studies in Epidemiology (STROBE) guidelines, and the STROBE checklist is included in Supplementary Table 1 available at https://doi.org/10.1192/bjo.2024.710. The population was individuals who underwent seclusion in a PICU. The exposures of interest were medication cooperation, seclusion duration, seclusion termination outside working hours, final seclusion review involving medical staff, insight into reasons for seclusion, and agitation or irritability in the 4 h prior to seclusion termination. The outcome was a composite of any of the following non-mutually exclusive events occurring within 24 h after the termination of seclusion: actual physical violence, attempted physical violence or reinitiation of seclusion.

### Setting

The study was conducted in South London and Maudsley NHS Foundation Trust, London, UK, which is the largest unit provider of secondary mental health services in the UK, providing local services to four London boroughs and specialist services nationally. The Trust has four PICUs (one female and three male), each accommodating one seclusion suite. The study used the Clinical Records Interactive Search (CRIS) system, which entails anonymisation of electronic healthcare records for subsequent research.^22^ The authors assert that all procedures contributing to this work comply with the ethical standards of the relevant national and institutional committees on human experimentation and with the Helsinki Declaration of 1975, as revised in 2008. CRIS is approved by the Oxfordshire C Research Ethics Committee (ref: 18/SC/0372), and this study was approved by the CRIS Oversight Committee (ref: 21–106). Under the terms of the ethical approval, informed consent is not required from the participants, but they are able to opt out of using their data for research.

During the preparation of this work the authors used Writefull Revise in order to improve the readability of the manuscript. After using this tool/service, the authors reviewed and edited the content as needed and take full responsibility for the content of the publication.

### Participants

Participants were included in the study if they were secluded in a PICU in the Trust and their seclusion was ended between 29 March 2018 and 4 March 2019. Seclusion episodes in forensic mental health units and health-based places of safety were excluded, as people in these facilities are referred for different reasons and may have distinct reasons for seclusion initiation and termination. Eligible seclusion episodes needed to have a seclusion record containing dates and times for seclusion initiation and termination; a match between structured fields and the free text to be able to ascertain additional details of the seclusion episode; and data on follow-up to 24 h after termination of seclusion.

### Variables

CRIS contains structured fields and free text. For data that were available in structured fields, this was extracted automatically. For variables that required review of the full text, authors (Gabriella L., M.L., C.W., A.M., F.H., R.G. and J.I.) examined the seclusion records and coded the records in a data extraction form. The outcome – any of actual physical violence, attempted physical violence or reinitiation of seclusion within 24 h of seclusion termination – was ascertained based on review of the free text for violent episodes and use of structured fields denoting a subsequent seclusion episode. These particular outcomes were chosen after discussion with clinicians working in PICUs because they were considered to be direct or indirect indicators of the perceived necessity of seclusion at a particular time. Other outcomes, such as self-harm, while very important for patients and clinicians, are not generally considered as legitimate reasons for the use of seclusion.^23^

The exposures were identified using the free text, apart from seclusion duration and seclusion termination out of hours, which were calculated using structured fields. Normal working hours were defined as Monday to Friday 09.00h–17.00h, excluding public holidays. A window of 4 h was used to define irritability or aggression, as medical reviews, which provide the most comprehensive assessment of mental state, were conducted on a 4-hourly basis. A full list of variables with their source and definition is provided in Supplementary Table 2.

### Sample size

A power calculation was conducted using G*Power 3.1.9.4. A logistic regression was planned assuming an alpha of 0.05 and power of 80%. The minimum difference that the study was powered to detect was between a probability of the outcome of 50% in one group and 30% in the other group. The sample size calculation estimated that 237 seclusion episodes would provide power of 80.1%. To account for potentially missing data or excluded cases, we increased this by 10% to 261 seclusion episodes.

### Statistical methods

A mixed-effects model was specified using seclusion episodes clustered within individuals. A hierarchical level could not be created for PICU because individuals could be secluded in more than one unit. A logistic regression was performed with a composite outcome of actual violence, attempted violence or seclusion reinitiation. Coefficients were exponentiated to provide odds ratios. In Model 1, exposures were entered into the model separately to assess univariable associations. In Model 2, PICU ward was added as a categorical fixed effect. In Model 3, additional covariates were added to Model 2, specifically ethnicity, age and a prior International Statistical Classification of Diseases and Related Health Problems, 10th revision (ICD-10) F2 diagnosis (Schizophrenia, and schizotypal and delusional disorders),^24^ as these predated the exposures and are plausible confounders. It was not possible to adjust for gender directly, as there was collinearity with the PICUs, which are designated as either male or female. Sensitivity, specificity, positive predictive value, negative predictive value, and area under the receiver operator characteristics curve of exposures that were statistically significant were calculated based on a fixed-effects model. In a secondary analysis, the covariates from Model 3 were added to Model 2 in turn to ascertain the impact of individual covariates. Missing data were handled using pairwise deletion (available case analysis); that is, for each statistical test, only those seclusion episodes where there were no missing data for that test were included. The analysis was conducted in *R* version 4.3.0 and RStudio 2023.03.0 with the ‘lme4’ package.^25^ Statistical significance was set to *P* < 0.05, and estimates were reported with 95% confidence intervals.

## Results

Data were extracted from the structured fields for 266 seclusion episodes. In all, 12 episodes were excluded (five duplicates, three where inspection of free text showed they were not actually in seclusion and four where it was not possible to link structured fields and free text). This left 254 episodes, representing 122 people, for whom full data extraction was performed. The number of seclusion episodes per individual ranged between one and eight. Forty individuals (32.8%) were female and 82 (67.2%) male. Ethnicity was Black in 85 (70.0%) individuals, White in 15 (12.3%), Mixed/Multiple in eight (7.6%), Other in 10 (8.2%) and unspecified in four (3.3%). All individuals were detained under the Mental Health Act. Descriptive statistics for the seclusion episodes are shown in Table 1.

**Table 1:** Descriptive statistics for seclusion episodes

	Variable	Statistics (*N* = 254)
Background demographic/clinical	Age at seclusion initiation/years, median (IQR)	29 (10)
	Primary ICD-10 diagnosis, *n* (%)	
	F2 – Schizophrenia, schizotypal and delusional disorders	166 (65.4)
	F3 – Mood [affective] disorders	51 (20.1)
	F6 – Disorders of adult personality and behaviour	6 (2.4)
	F8 – Disorders of psychological development	6 (2.4)
	F9 – Behavioural and emotional disorders with onset usually occurring in childhood and adolescence	13 (5.1)
	Not stated	12 (4.7)
	Any F2 diagnosis (primary or secondary), *n* (%)	171 (67.3)
	Legal status, *n* (%)	
	Detained under a civilian section of the Mental Health Act	248 (97.6)
	Detained under a forensic section of the Mental Health Act	6 (2.4)
	Informal (not detained)	0 (0)
	PICU, *n* (%)	
	Male unit 1	43 (16.9)
	Male unit 2	53 (20.9)
	Male unit 3	71 (28.0)
	Female unit	87 (34.3)
Seclusion-related	Seclusion duration/hours, median (IQR)	20.8 (38.7)
	Seclusion termination out of working hours, *n* (%)	138 (54.3)
	Type of terminating seclusion review, *n* (%)	
	Nursing	80 (31.5)
	Medical	134 (52.8)
	Not stated	40 (15.7)
	Reason for seclusion termination, *n* (%)	
	Clinically indicated	223 (87.8)
	Logistically necessary	29 (11.4)
	Not stated	2 (0.8)
	Debrief conducted with individual following seclusion, *n* (%)	
	Present	15 (5.9)
	Absent	234 (92.1)
	Not stated	5 (2.0)
Psychopathology	Insight into reason for seclusion, *n* (%)	107 (42.1)
	Agitation or irritability, *n* (%)	178 (70.1)
	Persecutory delusions, *n* (%)	91 (35.8)
	Control phenomena, *n* (%)	8 (3.1)
	Auditory hallucinations, *n* (%)	63 (24.8)
Prescribed medications	Long-acting injectable antipsychotic, *n* (%)	70 (27.6)
	Other antipsychotic, *n* (%)	160 (63.0)
	Antidepressant, *n* (%)	2 (0.8)
	Benzodiazepine, *n* (%)	201 (79.1)
	Mood stabiliser, *n* (%)	49 (19.3)
	Sedating antihistamine, *n* (%)	194 (76.4)
	Any non-cooperation, *n* (%)	88 (34.6)
Adverse outcomes within 24 h^a^	Actual physical violence, *n* (%)	50 (19.7)
	Attempted physical violence, *n* (%)	87 (34.3)
	Further seclusion, *n* (%)	32 (12.6)
	Any adverse outcome, *n* (%)	106 (41.7)

Where data were missing for a given variable, this is indicated by ‘Not stated’.

a. Outcomes were not mutually exclusive.

IQR, interquartile range; ICD-10, International Statistical Classification of Diseases and Related Health Problems, 10th revision; PICU, psychiatric intensive care unit.

The results of the mixed-effects logistic regression model examining the relationships between the exposures and the outcome are shown in Table 2. There was a statistically significant univariable relationship between agitation and irritability and the outcome with an odds ratio of 1.92 (1.03 to 3.56), *P* = 0.04, which remained similar after adjusting for specific PICU, ethnicity, age and prior F2 diagnosis (Schizophrenia, and schizotypal and delusional disorders).

**Table 2:** Results of mixed-effects model for predicting actual violence, attempted violence or seclusion reinitiation within 24 h of seclusion termination

Exposure	Model 1^a^		Model 2^b^		Model 3^c^	
	Odds ratio (95% CI)	*P*	Odds ratio (95% CI)	*P*	Odds ratio (95% CI)	*P*
Any medication non-cooperation	1.32 (0.74 to 2.35)	0.35	1.50 (0.82 to 2.73)	0.19	1.41 (0.77 to 2.58)	0.93
Seclusion duration/days	0.88 (0.73 to 1.05)	0.16	0.88 (0.73 to 1.06)	0.18	0.88 (0.73 to 1.07)	0.19
Seclusion termination out of working hours	1.39 (0.80 to 2.41)	0.25	1.37 (0.77 to 2.41)	0.28	1.40 (0.79 to 2.47)	0.25
Final seclusion review medical	1.01 (0.54 to 1.90)	0.98	1.04 (0.54 to 1.99)	0.91	1.03 (0.54 to 1.96)	0.93
Insight	0.79 (0.45 to 1.38)	0.40	0.71 (0.39 to 1.27)	0.24	0.71 (0.39 to 1.29)	0.26
Agitation or irritability	1.92 (1.03 to 3.56)	0.04	1.99 (1.04 to 3.81)	0.04	1.88 (0.98 to 3.62)	0.06

a. Univariable associations.

b. Model 1 with the addition of specific psychiatric intensive care units as fixed effects.

c. Model 2 with the addition of ethnicity, age and a prior F2 diagnosis (primary or secondary).

The odds ratios for the univariable associations between all covariates and the outcome are shown in Supplementary Table 3. The only statistically significant relationship was with F2 diagnosis (Schizophrenia, and schizotypal and delusional disorders), where the odds ratio was 0.53 (95% CI 0.29 to 0.98, *P* = 0.04). A secondary analysis with agitation/irritability as the exposure where the covariates in Model 3 were each added separately found that there was a minimal effect on the odds ratio; the full results are shown in Supplementary Table 4. The results of a sensitivity analysis in which the outcome was changed to actual violence or reinitiation of seclusion (not attempted violence) are shown in Supplementary Table 5. The odds ratios are similar to those shown in Table 2, though the confidence intervals are often wider, as the model has less power due to the use of a less common outcome.

Using a fixed-effects model for predicting an adverse outcome, the sensitivity of agitation or irritability was 77.4% (95% CI 68.2 to 84.9%), its specificity was 35.1% (95% CI 27.5 to 43.4%), the area under the receiver-operator characteristics curve was 0.563 (95% CI 0.507 to 0.618), the positive predictive value was 46.1% (95% CI 38.6 to 53.7%) and the negative predictive value was 68.4% (95% CI 56.7 to 78.6%). The raw figures are shown in Table 3.

**Table 3:** 2 × 2 table showing the prediction of adverse outcomes after seclusion termination of agitation/irritability

	Adverse outcome following seclusion termination		
	Present	Absent	Total
Agitation/irritability
Present	82	96	178
Absent	24	52	76
Total	106	148	254

## Discussion

In this retrospective cohort study, we investigated which factors predict an adverse outcome within 24 h after seclusion in a PICU is terminated. In 254 seclusion episodes from 122 people, we found that agitation or irritability in the 4 h prior to seclusion termination were associated with an approximately twofold increase in the odds of an adverse outcome (defined as actual violence, attempted violence or further seclusion). There was no statistically significant evidence to support a relationship between adverse outcomes and medication non-cooperation, seclusion duration, seclusion termination out of working hours, having a medical presence at a seclusion review or insight into the reasons for seclusion. However, the confidence intervals were broad, and we cannot rule out effect sizes that have some clinical significance.

There are several limitations to this work. While the study was premised on a sample size calculation that allowed us to detect very large effect sizes, there may be other predictors that are clinically significant that we were not powered to detect. This is suggested by some of the confidence intervals in Table 2. Moreover, given that we tested several hypotheses and our positive findings were only at the borderline of statistical significance, it is possible that they arose because of chance.

In terms of bias, there is likely to have been some misclassification in coding the free text due to unstructured, incomplete and ambiguous clinical entries. This misclassification is likely to be random and thus may have introduced a bias towards the null hypothesis. Psychopathology variables (for example, agitation or irritability) were not assessed systematically, so there is likely to have been subjectivity in recording by the clinicians and in interpretation of the notes by the researchers. In terms of confounding, it is likely that the relationship between some of the exposures and the outcome is confounded by illness severity. It is possible that this may have reduced any association with medical seclusion reviews, as confounding by indication might have meant that termination of seclusion for the most seriously unwell individuals was deferred to medical reviews.

Given that this study was conducted in one NHS Trust, in an area known to have a particularly high incidence of psychosis,^26^ the findings may have limited generalisability to other geographical regions. Moreover, the results cannot currently be generalised to seclusion in forensic settings or health-based places of safety. Furthermore, there have been changes in practice since 2019, including some driven by the COVID-19 pandemic, so further studies may be needed to investigate how this might impact our findings.

The main positive finding of this paper was that agitation or irritability in the 4 h prior to seclusion termination was associated with an increased risk of adverse outcomes after seclusion termination. This is consistent with the finding that irritability among psychiatric in-patients is associated with a substantially increased risk of violence or aggression in the following 24 h, resulting in its inclusion in the Dynamic Appraisal of Situational Aggression (DASA).^27^ This assessment tool has been found to have validity in populations with intellectual disability, male young offenders and individuals with personality disorders in high-security hospitals,^28–30^ although the irritability item may be less relevant to young offenders.^29^ Given that there is existing knowledge that would suggest agitation or irritability is a relevant factor, it is interesting to note that some individuals had their seclusion terminated despite the presence of agitation or irritability. Possible reasons include necessity (e.g. the seclusion room being required for another individual), agitation having been present within the previous 4 h but resolved at the point of termination, strategies being put in place to manage agitation out of seclusion, the presence of other reassuring features (e.g. knowledge of the individual, medication cooperation) or a perceived lack of importance of this clinical feature.

The negative findings included several factors that might be expected to be associated with an increased risk of adverse outcomes after seclusion termination. However, importantly, the confidence intervals for these exposures were often very wide, so we cannot exclude moderate effect sizes. For example, there are compelling reasons to think that staffing factors related to medical involvement in decision-making or termination of seclusion out of hours might be relevant; although these were not statistically significant in our model, we cannot rule out effect sizes with odds ratios as large as 1.90 or 2.41 respectively. It is interesting to note, however, that there is a lack of evidence to support stipulations of medical involvement in seclusion reviews,^23^ if it is for the purposes of predicting adverse outcome on seclusion termination.

In terms of clinical implications, our results suggest that clinicians should consider that agitation or irritability in the hours prior to possible seclusion termination may be associated with a higher risk of violence or further seclusion. However, there are important caveats surrounding its use in a clinical setting. Of particular note are the modest positive predictive value and negative predictive value, which are 46.1 and 68.4% respectively, suggesting that the absence of agitation or irritability may be more useful in predictive terms than its presence. Furthermore, our data-set is limited to those cases where clinicians were willing to end seclusion, so caution should be exercised in applying the results to cases where clinicians feel uncomfortable in ending seclusion. Therefore we suggest that the presence of agitation or irritability could be used as part of decision-making about the continuation of seclusion in situations where clinical teams are otherwise open to the prospect of terminating seclusion, being mindful of a broad consideration of risks and benefits.^14^ If seclusion is terminated despite the presence of agitation or irritability, clinical teams should consider being more vigilant for further aggression. More speculatively, clinical teams may anticipate in individuals with high agitation that ending seclusion safely may be problematic, so pharmacological or non-pharmacological interventions might be considered in advance of seclusion termination. In order to implement this, systematic assessment of agitation with a validated tool such as the DASA might be useful.

This study is important scientifically because, to our knowledge, it is the first to identify any factor that predicts adverse outcomes following termination of seclusion. It moves the field beyond anecdote and expert consensus to develop a systematic observational evidence base. It demonstrates the importance of the current mental state in this decision-making. Moreover, it shows that prediction over short periods of time in psychiatry is potentially a more tractable problem than the difficult field of prediction of risk of suicide and violence over months to years.

There are several future studies that could build on our work to create a more clinically useful literature. The most conceptually straightforward would be to replicate our study with a larger sample size. As we have mentioned, statistical power prevented us from establishing or ruling out moderate effect sizes for several theoretically interesting exposures in our study. Such a retrospective study could be augmented if clinical teams were routinely using validated instruments such as the DASA.^27^ A prospective study using structured data entry on a wide range of variables could go further by identifying factors that had not hitherto been conceived as potential predictors. Observational study designs would still be limited to cases where clinical teams thought seclusion could safely be ended, but – over time – variables identified with this approach could be applied and validated in cases of clinical equipoise.

## Supporting information

Rogers et al. supplementary material Rogers et al. supplementary material

## Data Availability

Data are owned by a third party, Maudsley Biomedical Research Centre (BRC) CRIS tool, which provides access to anonymised data derived from South London and Maudsley NHS Foundation Trust (SLaM) electronic medical records. These data can only be accessed by permitted individuals from within a secure firewall (i.e. the data cannot be sent elsewhere), in the same manner as the authors. For more information please contact: cris.administrator@slam.nhs.uk.

## Analytic code availability

The analytic code for the project is available on reasonable request from the corresponding author.
