# Supplementary material for: Identifying predictors of adverse outcomes after termination of seclusion in psychiatric intensive care units
Source: BJPsych Open. 2024 May 22;10(3):e120. doi: 10.1192/bjo.2024.710 (PMC11363086; doi:10.1192/bjo.2024.710)
Supplement: Rogers et al. supplementary material [file S2056472424007105sup001.docx]

# Supplementary material

## Contents

[Supplementary material 1](#_Toc138404098)

[Supplementary Table 1: STROBE checklist for cohort studies 1](#_Toc138404099)

[Supplementary Table 2: Properties of variables 2](#_Toc138404100)

[Supplementary Table 3: Univariable associations between covariates and outcome in a multilevel model 6](#_Toc138404101)

[Supplementary Table 4: Results of adding covariates separately to Model 2 for agitation-irritability 7](#_Toc138404102)

[Supplementary Table 5: Sensitivity analysis using actual violence or further seclusion within 24 hours as outcome 7](#_Toc138404103)

## Supplementary Table 1: STROBE checklist for cohort studies

|  | Item No | Recommendation | Location |
| --- | --- | --- | --- |
| **Title and abstract** | 1 | (*a*) Indicate the study’s design with a commonly used term in the title or the abstract | Abstract: Method |
|  |  | (*b*) Provide in the abstract an informative and balanced summary of what was done and what was found | Abstract: Method; Abstract: Results |
| Introduction | | |  |
| Background/rationale | 2 | Explain the scientific background and rationale for the investigation being reported | Introduction |
| Objectives | 3 | State specific objectives, including any prespecified hypotheses | Introduction |
| Methods | | |  |
| Study design | 4 | Present key elements of study design early in the paper | Methods: Study design |
| Setting | 5 | Describe the setting, locations, and relevant dates, including periods of recruitment, exposure, follow-up, and data collection | Methods: Setting |
| Participants | 6 | (*a*) Give the eligibility criteria, and the sources and methods of selection of participants. Describe methods of follow-up | Methods: Participants |
|  |  | (*b*) For matched studies, give matching criteria and number of exposed and unexposed | N/A |
| Variables | 7 | Clearly define all outcomes, exposures, predictors, potential confounders, and effect modifiers. Give diagnostic criteria, if applicable | Methods: Variables; Supplementary Table 2 |
| Data sources/ measurement | 8* | For each variable of interest, give sources of data and details of methods of assessment (measurement). Describe comparability of assessment methods if there is more than one group | Supplementary Table 2 |
| Bias | 9 | Describe any efforts to address potential sources of bias | Methods: Statistical methods |
| Study size | 10 | Explain how the study size was arrived at | Methods: Sample size |
| Quantitative variables | 11 | Explain how quantitative variables were handled in the analyses. If applicable, describe which groupings were chosen and why | Supplementary Table 2; Methods: Statistical methods |
| Statistical methods | 12 | (*a*) Describe all statistical methods, including those used to control for confounding | Methods: Statistical methods |
|  |  | (*b*) Describe any methods used to examine subgroups and interactions | Methods: Statistical methods |
|  |  | (*c*) Explain how missing data were addressed | Methods: Statistical methods |
|  |  | (*d*) If applicable, explain how loss to follow-up was addressed | N/A |
|  |  | (*e*) Describe any sensitivity analyses | Methods: Statistical methods |
| Results | | |  |
| Participants | 13* | (a) Report numbers of individuals at each stage of study—eg numbers potentially eligible, examined for eligibility, confirmed eligible, included in the study, completing follow-up, and analysed | Results |
|  |  | (b) Give reasons for non-participation at each stage | Results |
|  |  | (c) Consider use of a flow diagram | - |
| Descriptive data | 14* | (a) Give characteristics of study participants (eg demographic, clinical, social) and information on exposures and potential confounders | Table 1 |
|  |  | (b) Indicate number of participants with missing data for each variable of interest | Table 1 |
|  |  | (c) Summarise follow-up time (eg, average and total amount) | Methods: Study design |
| Outcome data | 15* | Report numbers of outcome events or summary measures over time | Table 1 |
| Main results | 16 | (*a*) Give unadjusted estimates and, if applicable, confounder-adjusted estimates and their precision (eg, 95% confidence interval). Make clear which confounders were adjusted for and why they were included | Table 2 |
|  |  | (*b*) Report category boundaries when continuous variables were categorized | Supplementary Table 2 |
|  |  | (*c*) If relevant, consider translating estimates of relative risk into absolute risk for a meaningful time period | N/A |
| Other analyses | 17 | Report other analyses done—eg analyses of subgroups and interactions, and sensitivity analyses | Supplementary Tables 3, 4, 5 |
| Discussion | | |  |
| Key results | 18 | Summarise key results with reference to study objectives | Discussion |
| Limitations | 19 | Discuss limitations of the study, taking into account sources of potential bias or imprecision. Discuss both direction and magnitude of any potential bias | Discussion |
| Interpretation | 20 | Give a cautious overall interpretation of results considering objectives, limitations, multiplicity of analyses, results from similar studies, and other relevant evidence | Discussion |
| Generalisability | 21 | Discuss the generalisability (external validity) of the study results | Discussion |
| Other information | | |  |
| Funding | 22 | Give the source of funding and the role of the funders for the present study and, if applicable, for the original study on which the present article is based | Funding |

## Supplementary Table 2: Properties of variables

| Variable category | Variable | Source | Definition | Pre-processing | Role in analysis | | | |
| --- | --- | --- | --- | --- | --- | --- | --- | --- |
|  |  |  |  |  | Baseline characteristic | Exposure | Covariate | Outcome |
| Demographic | Gender | Structured field | Binary (man 1, woman 0), as defined by the patient | - | ⚫ |  |  |  |
|  | Ethnicity | Structured field | Categorical as defined by the patient | Dichotomised as Black (largest ethnic group) and not Black | ⚫ |  | ⚫ |  |
|  | Age | Structured field | Integer denoting age / years at seclusion initiation | Used in decades for regression | ⚫ |  | ⚫ |  |
| Background clinical | PICU | Structured field | Categorical denoting which of 4 units (3 male, 1 female) the patient was secluded | - | ⚫ |  | ⚫ |  |
|  | Involuntary detention | Structured field | Binary denoting whether the person was detained under the MHA at the initiation of seclusion | - | ⚫ |  |  |  |
|  | MHA section | Structured field | Categorical denoting which section of the MHA a person was detained under | Dichotomised as civilian or forensic section | ⚫ |  |  |  |
|  | Diagnosis prior to seclusion | Structured field | Categoricals denoting the latest primary and secondary ICD-10 diagnoses prior to seclusion initiation  latest primary ICD-10 diagnosis prior to seclusion initiation | For baseline characteristics, primary diagnosis grouped within F categories (e.g. F2, F3). For analysis, grouped as any (primary or secondary) F2 diagnosis (schizophrenia, schizotypal and delusional disorders) vs no F2 diagnosis. | ⚫ |  | ⚫ |  |
|  | Seclusion duration | Structured field, checked against free text | Numeric denoting time from seclusion initiation to seclusion termination. Occasionally, there was an error in coding the start of the seclusion and this was correct with reference to the full text. |  | ⚫ | ⚫ |  |  |
|  | Seclusion termination out of hours | Structured field, checked against free text | Binary denoting whether seclusion episode was terminated outside normal working hours. Normal working hours defined as Monday to Friday 09:00 – 17:00, excluding public holidays. |  | ⚫ | ⚫ |  |  |
|  | Seclusion termination clinically indicated | Free text | Binary denoting whether seclusion termination was judged to be clinically indicated (i.e. the right decision for the patient) [1] or was necessitated by other circumstances (e.g. seclusion suite required for another patient, problem with physical environment) [0] | - | ⚫ |  |  |  |
|  | Evidence for a debrief with the patient following seclusion termination | Free text | Binary denoting whether a discussion was held with the patient about seclusion after seclusion had been formally terminated | - | ⚫ |  |  |  |
|  | Type of final seclusion review | Free text | Binary denoting whether the review was nursing-led [0] or medical-led [1]. | - | ⚫ | ⚫ |  |  |
| Medication-related (unless otherwise stated, these refer to the entire seclusion period) | Pre-existing antipsychotic LAI | Free text | Binary denoting whether at the time of seclusion initiation the patient was taking an antipsychotic LAI | - | ⚫ |  |  |  |
|  | Antipsychotic offered | Free text | Binary denoting whether an antipsychotic medication (other than a LAI) was offered | - | ⚫ |  |  |  |
|  | Antidepressant offered | Free text | Binary denoting whether an antidepressant medication was offered | - | ⚫ |  |  |  |
|  | Benzodiazepine offered | Free text | Binary denoting whether a benzodiazepine was offered | - | ⚫ |  |  |  |
|  | Mood stabiliser offered | Free text | Binary denoting whether a mood stabiliser was offered | - | ⚫ |  |  |  |
|  | Promethazine offered | Free text | Binary denoting whether promethazine was offered | - | ⚫ |  |  |  |
|  | Medication adherence | Free text | Ordinal (none, partial, full) denoting the extent to which the patient was voluntarily adherent with medications during seclusion. | Dichotomised to a binary variable (any-noncompliance, full compliance) | ⚫ | ⚫ |  |  |
| Psychopathology | Agitation or irritability prior to seclusion termination | Free text | Binary denoting whether agitation was present in the 4hr prior to seclusion termination | - | ⚫ | ⚫ |  |  |
|  | Persecutory delusions prior to seclusion termination | Free text | Binary denoting whether persecutory delusions were present in the 4hr prior to seclusion termination | - | ⚫ |  |  |  |
|  | Control phenomena prior to termination | Free text | Binary denoting whether control phenomena were present in the 4hr prior to seclusion termination | - | ⚫ |  |  |  |
|  | Auditory hallucinations prior to termination | Free text | Binary denoting whether auditory hallucinations were present in the 4hr prior to seclusion termination | - | ⚫ |  |  |  |
|  | Insight around reason for seclusion | Free text | Ordinal (absent, partial, present) denoting whether the patient had insight for the reason for seclusion within the 4hr prior to seclusion termination | Dichotomised to a binary variable (any insight, no insight) | ⚫ | ⚫ |  |  |
| Outcome | Actual physical violence after seclusion termination | Free text | Binary denoting whether the patient engaged in actual physical violence in the 24hr after seclusion termination | Combined with attempted physical violence and further seclusion event |  |  |  | ⚫ |
|  | Attempted physical violence after seclusion termination | Free text | Binary denoting whether the patient attempted physical violence in the 24hr after seclusion termination | Combined with actual physical violence and further seclusion event |  |  |  | ⚫ |
|  | Further seclusion event | Structured field. (Occasionally, where there was conclusive evidence from the free text that there was an error in the structured field, this was amended to match the free text.) | Binary denoting whether the patient re-entered seclusion within 24hr of the previous seclusion terminating | Combined with actual physical violence and attempted physical violence |  |  |  | ⚫ |

LAI = long-acting injection

## Supplementary Table 3: Univariable associations between covariates and outcome in a multilevel model

| Covariate | OR (95% CI) | *p* |
| --- | --- | --- |
| PICU |  |  |
| - Female PICU (reference) | - | - |
| - Male PICU 1 | 0.82 (0.34 to 2.01) | 0.67 |
| - Male PICU 2 | 1.58 (0.70 to 3.57) | 0.27 |
| - Male PICU 3 | 0.62 (0.28 to 1.34) | 0.22 |
| Black ethnicity | 0.76 (0.38 to 1.51) | 0.43 |
| Age / decades | 1.06 (0.74 to 1.53) | 0.75 |
| F2 diagnosis | 0.53 (0.29 to 0.98) | 0.04 |
| F3 diagnosis | 0.98 (0.44 to 2.16) | 0.95 |
| Seclusion termination for logistical reasons | 1.70 (0.73 to 3.94) | 0.21 |
| Seclusion debrief conducted | 1.26 (0.40 to 3.93) | 0.69 |
| Persecutory delusions present | 1.17 (0.65 to 2.07) | 0.60 |
| Control phenomena present | 0.78 (0.16 to 3.74) | 0.76 |
| Auditory hallucinations present | 0.94 (0.50 to 1.79) | 0.86 |
| Long-acting injectable antipsychotic used | 0.58 (0.30 to 1.14) | 0.12 |
| Other antipsychotic offered | 1.06 (0.60 to 1.86) | 0.84 |
| Benzodiazepine offered | 1.20 (0.61 to 2.36) | 0.60 |
| Mood stabiliser offered | 0.79 (0.38 to 1.64) | 0.52 |
| Sedating antihistamine offered | 0.60 (0.31 to 1.15) | 0.12 |

PICU = psychiatric intensive care unit

## Supplementary Table 4: Results of adding covariates separately to Model 2 for agitation-irritability

| Model | OR (95% CI) | *p* |
| --- | --- | --- |
| Model 2: agitation-irritability and PICU | 1.99 (1.04 to 3.81) | 0.04 |
| Model 2 + ethnicity | 2.01 (1.04 to 3.88) | 0.04 |
| Model 2 + age | 1.97 (1.03 to 3.79) | 0.04 |
| Model 2 + F2 diagnosis | 1.90 (1.00 to 3.62) | 0.05 |

PICU = psychiatric intensive care unit

## Supplementary Table 5: Sensitivity analysis using actual violence or further seclusion within 24 hours as outcome

| Exposure | Model 1 ^a^ | | Model 2 ^b^ | | Model 3 ^c^ | |
| --- | --- | --- | --- | --- | --- | --- |
|  | OR (95% CI) | *p* | OR (95% CI) | *p* | OR (95% CI) | *p* |
| Any non-compliance | 1.23 (0.66 to 2.27) | 0.52 | 1.31 (0.70 to 2.45) | 0.40 | 1.26 (0.67 to 2.34) | 0.47 |
| Seclusion duration (days) | 0.91 (0.75 to 1.11) | 0.37 | 0.94 (0.77 to 1.15) | 0.54 | 0.94 (0.77 to 1.15) | 0.54 |
| Seclusion termination out of working hours | 1.82 (0.99 to 3.34) | 0.05 | 1.73 (0.93 to 3.21) | 0.08 | 1.77 (0.96 to 3.28) | 0.07 |
| Final seclusion review medical | 0.72 (0.38 to 1.39) | 0.33 | 0.74 (0.39 to 1.43) | 0.38 | 0.73 (0.38 to 1.40) | 0.34 |
| Insight | 0.59 (0.32 to 1.10) | 0.10 | 0.60 (0.32 to 1.14) | 0.12 | 0.59 (0.31 to 1.12) | 0.10 |
| Agitation or irritability | 1.93 (0.96 to 3.89) | 0.07 | 1.83 (0.89 to 3.76) | 0.10 | 1.80 (0.88 to 3.68) | 0.11 |

^a^ Univariable associations. ^b^ Model 1 with the addition of specific psychiatric intensive care units as fixed effects. ^c^ Model 2 with the addition of ethnicity, age and a prior F2 diagnosis (primary or secondary).
